# Barriers and facilitators for breast cancer early diagnosis in an indigenous community in Mexico: voices of *otomí* women

**DOI:** 10.1186/s12905-023-02875-2

**Published:** 2024-01-13

**Authors:** Minerva Saldaña-Téllez, Sergio Meneses-Navarro, Leonor Cano-Garduño, Karla Unger-Saldaña

**Affiliations:** 1 COMECyT (Council of Science and Technology of State of Mexico), Toluca, Mexico; 2 CONAHCYT (National Council of Science and Technology)-Center for Research in Health Systems, National Institute of Public Health, Mexico City, Mexico; 3 CEDIPIEM (Center for the Development of the Indigenous People of the State of Mexico), Mexico City, Mexico; 4 CONAHCYT (National Council of Science, Humanities and Technology), National Cancer Institute, Mexico City, Mexico

**Keywords:** Early diagnosis, BC, Indigenous women, Otomí population, Mexico

## Abstract

**Background:**

Literature on barriers and facilitators for early detection of Breast Cancer (BC) among indigenous women is very scarce. This study aimed to identify barriers and facilitators for BC early diagnosis as perceived by women of the *otomí* ethnic group in Mexico.

**Methods:**

We performed an exploratory qualitative study. Data was collected in 2021 through three focus group interviews with 19 *otomí* women. The interview transcripts were analyzed using the constant comparison method and guided by a conceptual framework that integrates the Social Ecological Model (SEM), the Health Belief Model and the Institute of Medicine’s Healthcare Quality Framework.

**Results:**

Barriers and facilitators were identified at several levels of the SEM. Among the main barriers reported by the study participants were: beliefs about illness, cancer stigma, cultural gender norms, access barriers to medical care, and mistreatment and discrimination by health care personnel. Our participants perceived as facilitators: information provided by doctors, social support, perceived severity of the disease and perceived benefits of seeking care for breast symptoms.

**Conclusions:**

Healthcare policies need to be responsive to the particular barriers faced by indigenous women in order to improve their participation in early detection and early help-seeking of care for breast symptoms. Measures to prevent and eradicate all forms of discrimination in healthcare are required to improve the quality of healthcare provided and the trust of the indigenous population in healthcare practitioners.

## Background

Breast Cancer (BC) is the most common type of cancer among women worldwide. Even though BC incidence is higher in high-income countries in comparison to low- and middle-income countries (LMICs), the majority of deaths actually occur in LMIC settings [1] The higher mortality rates observed in LMICs compared to high-income countries (HICs) are thought to be a consequence of late detection and limited access to standard quality treatment [1].

In Mexico, BC is the most frequent cancer and the main cause of cancer mortality among women since 2006 [2]. The majority of BC cases (65%) are diagnosed at advanced stages (IIB to IV) and the estimated overall survival is 72% [3]. Although the burden of BC disease is usually higher in urban populations, incidence has been also increasing in Mexico’s rural populations [4]. Evidence shows that women living in marginalized areas have a higher risk of dying from preventable cancer deaths than other populations due to a combination of vulnerabilities that often result in late detection and delayed or incomplete treatment [5–7].

Indigenous minorities in Mexico compound several vulnerabilities: they tend to have lower socioeconomic status, less access to education, and more commonly live in small rural communities that lack access to many services, including healthcare [7–10]. Gender and ethnicity interact and indigenous women in Mexico face a double social vulnerability: that of being women in a society where power structures favor men and that of belonging to a minority ethnic group that has suffered systemic discrimination for more than 200 years [11, 12]. Indigenous women in Mexico experience the greatest lags in health (e.g., the lowest life expectancy at birth and the highest maternal and infant mortality ratios), and face the greatest barriers to accessing health services including discrimination at healthcare facilities [13, 14]. The superimposition or intersectionality of these social factors of vulnerability configure the systematic inequalities that determine the subordinate position of indigenous women in the social structure [15].

Several barriers have been described in the international literature for early BC diagnosis among minority populations living in HICs, like immigrants and, afro-descendants [16–20]. However, there is a dearth of studies related with barriers and facilitators of BC early diagnosis in indigenous populations worldwide. In Mexico, the scarce existing literature on barriers and facilitators for early detection of cancer among indigenous women has been limited to understanding their participation in cervical cancer screening [21–23]. Therefore, we undertook this qualitative study to explore barriers and facilitators for early BC diagnosis as perceived by *otomí* women living in the suburbs of an urban city in central Mexico.

## Methods

### Design

An exploratory and descriptive qualitative study was conducted [24, 25] with *Otomí* women living in Jiquipilco, State of Mexico. The study received approval from the National Cancer Institute of Mexico’s institutional review boards (021/041/IBI) (CEI/1592/21).

### Study setting

The State of Mexico is a neighbor state of Mexico City. Jiquipilco is located approximately 45 km from Toluca, the capital of the state, and has a population of 69,031 habitants, of which 23.2% identify as indigenous [26]. It has an urban central area surrounded by rural areas, and its main economic activity is agriculture.

The *Otomí* people are one of the original ethnic groups of Mexico and live across different regions of the country [27]. In the State of Mexico, the *Otomí* population concentrates in 21 municipalities, and Jiquipilco is one of them. In Jiquipilco, the *Otomí* people tend to concentrate in the rural outskirts of the municipality, living in conditions of poverty, and limited access to services, education and employment opportunities [10, 27, 28]. The *Otomí* people of the State of Mexico tend to work in agriculture activities part of the year, mainly in the cultivation of corn, beans, wheat, oats and maguey [27]. In the months when there is no agricultural activity, they migrate from rural communities to the Metropolitan Areas of Toluca and Mexico City where they are most employed as domestic workers, peddlers or as construction workers [27, 29]. These occupations are in the informal sector of the economy, and therefore most *Otomí* people are not covered by social security health insurance, which is provided through formal employment in Mexico. National Guidelines for BC Control in Mexico recommend: monthly breast self-examination starting at age 20, annual clinical breast examination (CBE) starting at age 25, and screeningmammography every 2 years starting at age 40 and up to 69 years [30]. Access to CBE and screening mammography varies according to women’s health insurance coverage and their capacity to pay for private services. At the time of this study, approximately 40% of the National population was covered by a social security health insurance scheme and only 3% had private insurance. For the uninsured, the state offers health services through its own infrastructure. Mammography units closest to Jiquipilco are in the state capital (Toluca), which is approximately 45 km from Jiquipilco.

### Study participants

We used intentional non-probability sampling to find adult Otomí women -in Mexico, legally adulthood starts at 18 years of age- native of and currently living in Jiquipilco who could speak Spanish, and had no personal history of breast cancer [24]. The main objective of intentional sampling is to elicit different perspectives from people who represent the opinion of their group of reference [31, 32]. The vast majority of indigenous people who live in Jiquipilco speak Spanish. According to data from the National Census, in Jiquipilco 8.0% of residents speak an indigenous language, and only 0.1% of the population speaks an indigenous language and no Spanish [33]. The State’s Council for the Integral Development of Indigenous Peoples (CEDIPIEM for its acronym in Spanish) helped us establish contact with the community to facilitate the invitation of potential participants. CEDIPIEM is a decentralized public entity whose purpose is to define, execute and evaluate policies directed to improve the lives of the State of Mexico’s indigenous population [27]. Even though, inviting participants through an official organization could have increased the risk of selection bias, this was the best alternative we found to identify and invite otomí women of the region who would trust our invitation. We explained the study to all of those invited, emphasizing that participation was voluntary and that there would be no repercussions on health care or social benefits if they refused to participate. Written informed consent was signed by all participants previous to their participation in the focus group interviews. We included all who were willing to participate.

### Conceptual framework

**Fig. 1:**
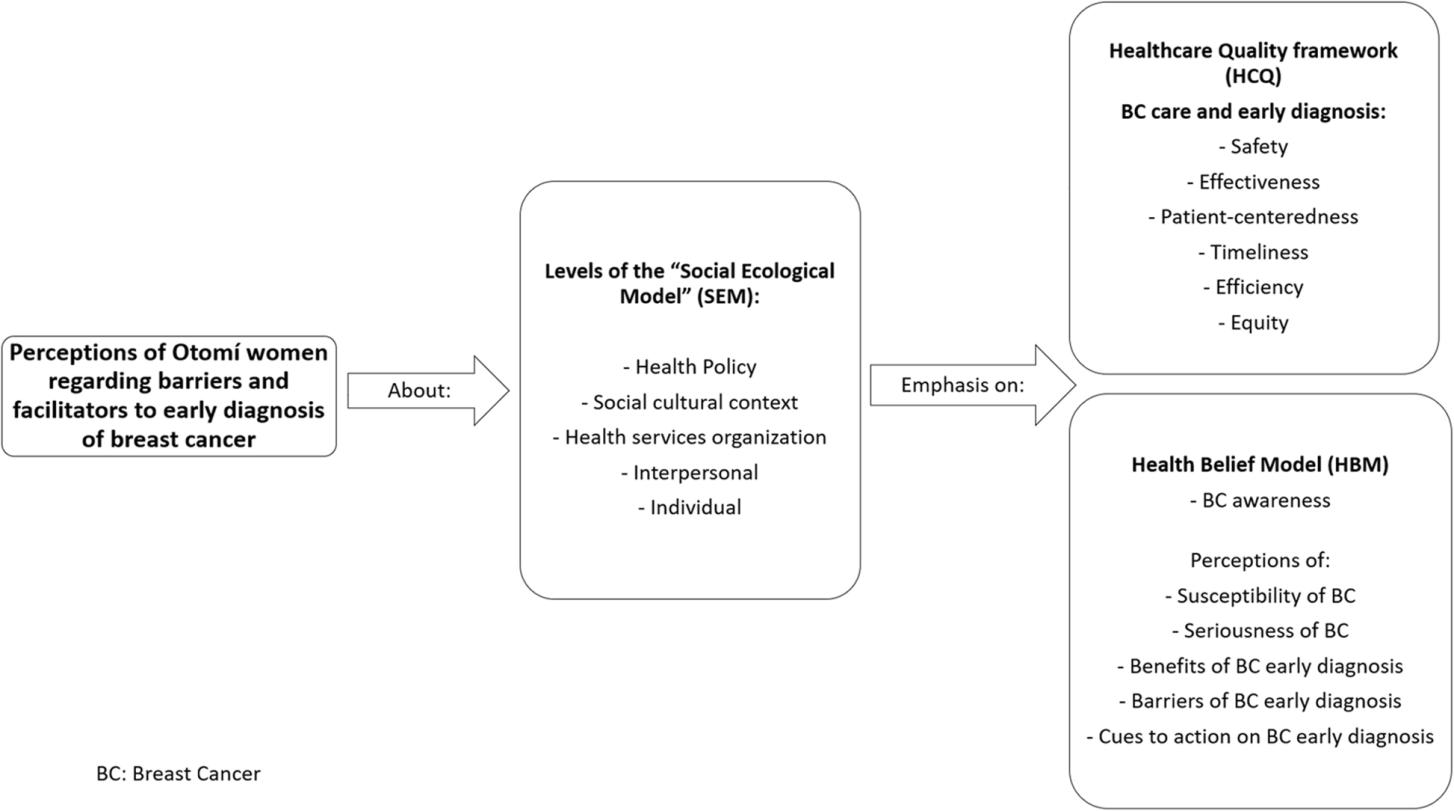
Conceptual framework that guided our Interview Analysis

Our study was guided by a conceptual framework that integrates the Social Ecological Model [34], the Health Belief Model [35] and the Institute of Medicine’s Healthcare Quality framework [36]. Figure 1 illustrates how we integrated these three theoretical perspectives to guide our interview analyses in the identification of the Otomí women’s perceived barriers and facilitators for early diagnosis of BC.

The Social Ecological Model (SEM) is a useful framework to identify the full range of factors that can influence health and health behavior. These factors can be located at different levels: individual, interpersonal, institutional or organizational, community, and public policy levels. The SEM framework emphasizes the interaction and interdependence between factors within and across all these levels [34, 37, 38]. It has been used to study diverse social problems and health behaviors [39–45].

The SEM can be used to integrate components of other theories. We used the Health Belief Model (HBM) to strengthen the analysis of individual level factors that exert an influence on *Otomí* women’s help seeking behavior, and the Healthcare Quality (HCQ) framework to strengthen our analysis of the organizational level factors (our participants’ perception of the quality of services for BC early diagnosis: primary care clinics and breast imaging services).

The HBM stipulates that the following groups of factors influence the likelihood of a person taking a recommended preventive health action: demographic variables -age, race, socioeconomic level-; psychological variables and knowledge of disease (in this case, BC); perceptions of the disease (perceived susceptibility and perceived seriousness of BC); perceptions of the health behavior of interest (perceived benefits and perceived barriers to act on the recommended health behavior) and cues to action [35]. Perceived susceptibility refers to a person’s subjective perception of their own risk of developing BC. Perceived severity includes assessments of severity and the medical and social consequences of getting BC. Perceived barriers refer to the possible negative effects of the preventive or health behavior such as its costs, secondary adverse effects, and time required. Perceived benefits refer to the individual’s perception of the effectiveness of the health behavior. The main health behavior we were interested in understanding was timely seeking of medical care for breast symptoms, but we also assessed the study participants’ perceived benefits of breast self-examination, screening clinical breast examination and screening mammography. Finally, cues to action are events or things that trigger people to act or perform a certain health behavior (e.g., medical recommendation, mass media messages, etc.) [46]. Over time this model evolved to include self-efficacy as an important determinant in health behavior [47]. Self-efficacy is understood as the conviction of people in their own capability to successfully perform a certain behavior [48].

Finally, we used the Health Care Quality (HCQ) framework to strengthen our analysis of the health system (organizational level) factors. According to the HCQ framework, quality healthcare should be: 1) safe, avoiding harm to patients from the care that is intended to help them, 2) effective, providing services based on scientific knowledge to all who could benefit and refraining from providing services to those not likely to benefit, 3) patient-centered: providing care that is respectful of and responsive to individual patient preferences, needs, and values and ensuring that patient values guide all clinical decisions, 4) timely: reducing waits and sometimes harmful delays for both those who receive and those who give care, 5) efficient: avoiding waste, including waste of equipment, supplies, ideas, and energy, and 6) equitable: providing care that does not vary in quality because of personal characteristics such as gender, ethnicity, geographic location, and socioeconomic status [36]. Even though we did not analyze patient nor services outcomes, using this framework we were able to identify our participants’ perceptions on HCQ dimensions based on their previous interactions with health services. In this study, we were particularly interested in our participants’ previous experiences with health services and their perceptions regarding patient-centeredness, as this can be especially challenging in the context of care for women that belong to a historically marginalized and discriminated social group.

### Data collection

We conducted three focus group interviews with 19 women in November 2021 [49]. Focus group interviews are recognized as a useful tool to obtain information about collective points of view and their meanings, and to generate a rich understanding of the experiences and beliefs of the participants [50]. MST moderated the interviews. She is a woman, psychologist and qualitative researcher with no previous relationship with the Otomí community at Jiquipilco nor with the CEDIPIEM.

The interviews were conducted using a semi-structured interview guide with open-ended questions to ask participants about their perceptions of barriers and facilitators, knowledge, attitudes and beliefs about cancer early detection in general and more specifically about early BC diagnosis. We developed our interview guides based on our conceptual framework and key findings from the existing literature on barriers and facilitators for early detection of BC among underserved populations.

Each focus group interview lasted approximately 60 minutes and the number of participants in the groups ranged between 4 and 8. All interviews were audio-recorded. Data saturation was achieved with the last focus group and, therefore, no more participants were recruited. We decided saturation was reached when no new codes appeared and each of the codes had been applied to a sufficient amount of data [49, 51]. We also collected descriptive demographic data from all the participants including age, marital status, occupation, years of school education and family income.

### Data analysis

Participants’ responses were transcribed verbatim and all transcripts were de-identified prior to analysis. Transcripts and field notes were organized using Atlas.ti 8 software to aid the analysis. We used a pragmatic approach for data interpretation, using both deductive and inductive data analysis to explain findings. These type of analytical processes that engage both deductive and inductive strategies have shown to help researchers apply concepts from the literature and theory, which can in turn support the trustworthiness and applicability of the study [52]. We identified barriers and facilitators for early BC diagnosis guided by our conceptual framework (Fig. 1) which integrates theoretical perspectives of the Social Ecological Model, the Health Belief Model and the Institute of Medicine’s HealthCare Quality Framework [53, 54]. But data was also coded using the constant comparison method. The constant comparison method is an iterative and inductive process of reducing the data through constant recoding to assure that all data are systematically compared to all other data in the data set [55, 56]. Using this strategy we continually compared data to other data within a single interview, between interviews within the same group and between interviews from different groups [57]. We read all interview transcripts carefully several times in order to identify the codes through the participants’ narratives. To enhance trustworthiness and rigor, we used triangulation for coding of the data. Data were coded by two different researchers: MST who is psychologist with postgraduate studies in health psychology and KUS is a medical doctor and health systems researcher. The coding results were then reviewed for cases with differing results, reaching consensus between the two coders to establish the final codes.

## Results

Nineteen Otomí women participated in the study. To keep the confidentiality agreement we made with all of our participants, the names used in this paper are pseudonyms. Participant sociodemographic characteristics are shown in Table 1**.**

**Table 1:** Sociodemographic characteristics

Characteristics		No.
Age *(Mean, range)*		*37 (18–61)*
Marital status	In a relationship	6
	Not in a relationship	13
Occupation	Unemployed	10
	Employed	8
	No response	1
Education	6 years or less	3
	7 to 9 years	7
	10 years or more	9
Otomí speakers	Yes	9
	No	10
Health insurance	Yes	9
	No	10
Monthly family income^a^	< 1 minimum salary	14
	1- < 3 minimum salaries	2
	≥3 minimum salaries	3

^a^1 minimum monthly salary ~ 207 USD as defined by the Mexican government in 2021

**Fig. 2:**
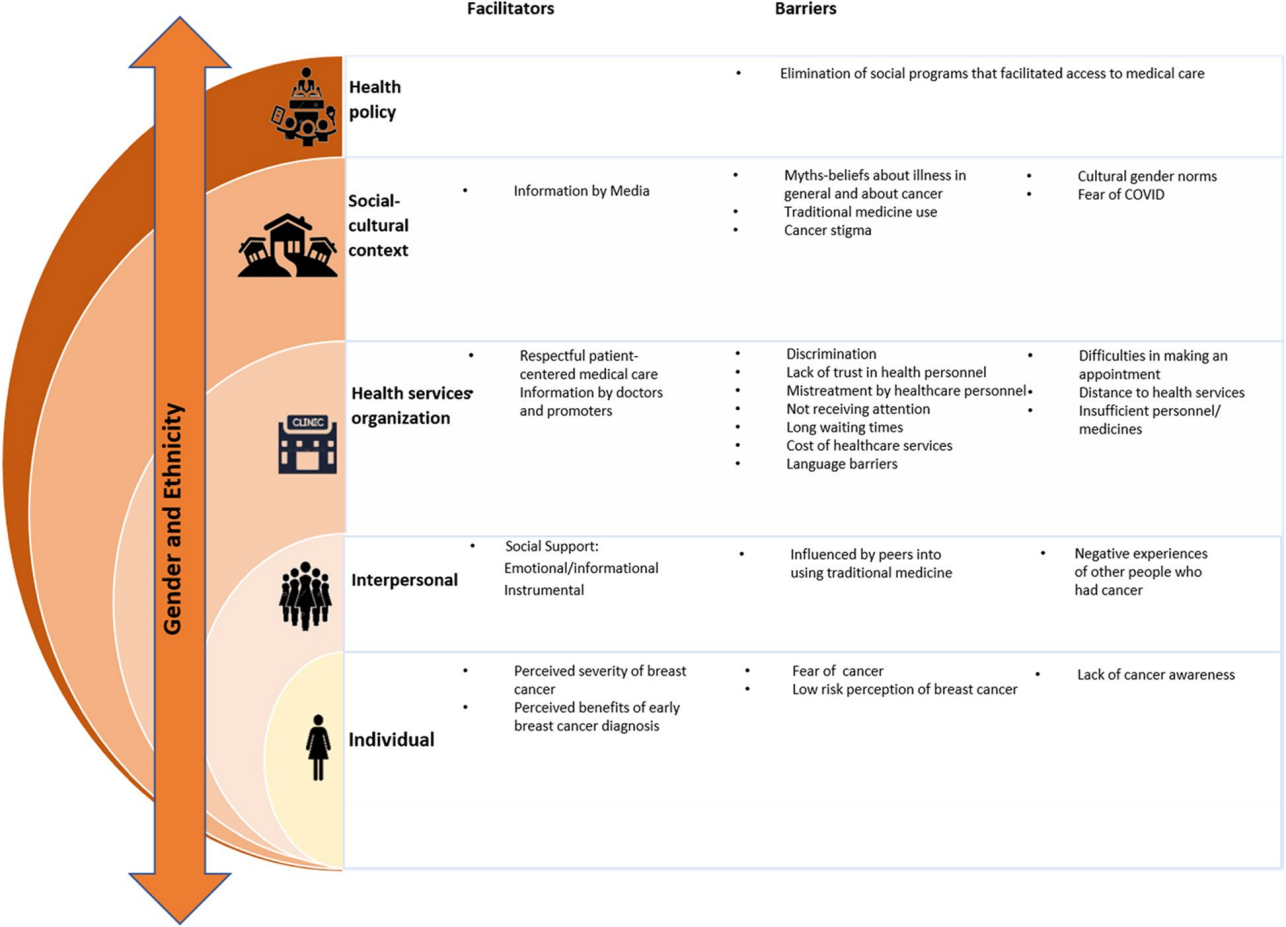
Perceived barriers and facilitators at different levels of the SEM

Figure 2 summarizes the perceived barriers and facilitators for BC early diagnosis that we identified in the interviews, and organizes them at the different levels of the Social Ecological Model (SEM). The Health Belief Model constructs were used to code the barriers and facilitators identified at the individual level of the SEM, and the Healthcare Quailty framework was used for the Health Services Organization level. The arrow crossing through all levels represents gender and ethnicity as the key social processes that act at every level of the SEM to influence individual women’s help-seeking behaviors for breast symptoms and timely access to quality medical care for BC early diagnosis.

### Perceived barriers to early BC diagnosis

#### Health policy barriers

Our study participants perceived the elimination of the social program “*Progresa-Oportunidades-Prospera*” (POP) as an access barrier to healthcare services. This was a federal program that gave conditional cash transfers to families living in poverty to improve their access to nutritional food, healthcare and education. The program operated for 20 years and was terminated in 2019 by the current government [53]. The POP program provided basic health services free of charge, in addition to health promotion actions under three modalities: self-care promotion; individualized guidance and counseling during medical consultations; and health promotion messages aimed at the families of beneficiaries [58].

Our interviewees reported that through *POP* they had access to special health programs, health information and better access to health care. They perceive that, as a result of its elimination, people seek less care at health centers, as they report experiences of not receiving medical attention at the health center when they need it, and they feel “lost” regarding where to seek medical attention.

*“…When we had the program, well, that program worked in conjunction with the health center, so the nurses gave us health talks and workshops and taught us how to examine ourselves. Yes, in fact that program was very good, because they also taught us things like healthy eating, how to exercise…”* (María 52 years old).

“*Since the program disappeared… we no longer have the same attention and in fact, I feel proud of those years, when that program existed…because in those years I went with the doctors and they provided medical care, when they realized that the pain did not decrease, they gave me a referral to see a specialist, a gynecologist, and that’s where my myomas were diagnosed…”* (Sandra, 41 years old).

*“…Everything changed, now we feel like we’re lost, like we don’t even know where to go, with what doctor we can go. Before, when the program existed, there were members here in the community that organized people and would take them to health workshops and to get medical attention in health centers…”* (Teresa, 47 years old).

#### Social and cultural context barriers

##### Cultural gender norms

Gender issues constantly emerged in the participants’ narratives. Women spoke about cultural gender norms and men’s attitudes towards sexuality as a barrier to BC early diagnosis. They referred to men as being “*machista*”, trying to control their female partners’ behavior. They explained that in their community it was prohibited for women to talk about their breasts, to examine their own breasts, and to get general check-ups with male doctors.

*“…If the husbands find them touching their breasts...they question them “why are you doing that? you can’t do that, why are you touching yourself?” I feel like that is machismo… You cannot touch and explore your breasts without being sexualized…”* (Cecilia, 28 years old).

*“…Yes, oh yes, there are many men who do not let women go to the doctor, that kind of men predominate here. That’s because in our community there is machismo…”* (Teresa, 47 years old).

Our participants described that this “sexual taboo” limits them to talk about their bodies, their breasts and breast diseases because they feel embarrassed. They said that they do not know their own bodies and that they don’t explore their breasts because of shame and fear of being judged.

*“…So, the context has a lot of influence here, I feel that this community is very conservative, for example, I don’t talk about those things (breast topics) because it is frowned upon, and I know it could be misunderstood ...”* (Margarita, 38 years old).

Due to the assigned gender roles in the community, girls receive less school education than boys. Our participants reported that once girls finish the mandatory 6 years of elementary school education in Mexico, they are considered to be ready for marriage. These low levels of schooling not only have a negative impact in indigenous women’s health literacy and awareness of different health problems, like cancer, but also in their own empowerment to fight for their rights within their families, their communities and in their exchanges with healthcare services.

Additionally, as part of their gender roles, women in the community are expected to take care of their children, spouse, and other family members, prioritize the care of others over their self-care, and are also responsible for all the housework (buying food, cooking, cleaning, washing clothes, etc.). They usually have several children as they are not empowered to negotiate birth control with their partners. More and more women are also working outside the house, in search for better economic conditions, but the gender roles of taking care of others and the household are still in place. Our participants referred that they hardly have any time to take care of themselves and this makes it very difficult to seek healthcare when they feel ill and even more so for preventive activities.

*“...I feel that we always have time for everything, except for our health, for example I invited some women to a health talk and they did not go… I think we don’t take care of ourselves, we don’t go to the doctor, always the family first, always the children, always the house, always! What about us? We are always last…”* (Verónica, 46 years old).

##### Myths and beliefs about illness in general and about cancer

Beliefs about illness in general were also perceived as barriers for early diagnosis of BC by our participants. For example, they believe that if they think about a certain disease, they can attract it and then fall ill. For this reason, people in the community tend not to talk or think about diseases, as they believe that this way they will avoid getting sick. This makes it very difficult for people in the community to be willing to get health information and to participate in preventive and early detection behaviors.

*“… Sometimes we psychologically call disease, so we better not think about it, we better think that it is far away and is not going to touch us, so we don’t get sick…”* (Nancy, 50 years old).

Another common belief about illness in this community, as described by our participants, is that they only perceive themselves to be ill when they feel that their life is in danger. Additionally, they seek medical care only if they feel ill or interpret their symptoms as being life-threatening.

*“…The people of my community, well, no, they don’t go to the doctor, well I think we all go to the doctor until we feel a lot of pain, as we said, when the problem is already very advanced. I had a neighbor who got sick with cancer, the cancer attacked several systems, organs and she died because nothing could be done, not even with chemo, she went to the doctor too late…”.* (Cecilia, 28 years old).

##### Cancer stigma

Cancer stigma was perceived as a barrier to seeking medical care. Some participants reported reluctance to talk about cancer in their community and commented that women with BC generally do not reveal their diagnosis even to their own families. They believe this is because of the common belief that cancer is a consequence of having misbehaved, “having been bad”. They see cancer as a divine punishment, so people avoid sharing their diagnosis because of fear of feeling judged by their family members and friends.

*“…Sometimes, we are embarrassed to say we have a disease, we don’t want our neighbors or other people to know. We think that they will judge us, that people will say “if she’s sick, it’s because she surely did something bad and God punished her”…That’s why people don’t say anything when they feel ill... About women with BC or with cervical cancer, many times people say: “she did something wrong, God punished her for that”... It’s better we don’t talk, it’s better we don’t say we’re sick...”* (Margarita, 38 years old).

Additionally, in regard to BC, they spoke about the stigma in relation to mastectomy and “being a woman without breasts”.

*“…It is complicated because cancer “eats you from the inside”, to the point that the entire breast has to be amputated, they have to remove the breast. No, the word is not removal, it is amputation, they amputate the entire breast, which is sometimes hard and difficult to assimilate, imagine a woman without breasts…”* (Nancy, 50 years old).

Cancer in general is viewed as a fatal disease, which they associate with death, pain, suffering and aggressive treatments. Therefore, if they think their symptoms are related with cancer, they are likely to postpone seeking medical care in order to avoid what they see as aggressive unnecessary treatments. This belief is further confirmed once people seek care very late and so in fact receive aggressive treatments and nevertheless die soon.

##### Traditional medicine use

Also, our participants said that sometimes people in their community prefer using traditional medicine and postpone seeking medical care, or interrupt medical treatment in favor of traditional medicine treatment.

*“…Well, a neighbor of my community was going with a “healer” who is, according to her, very famous for healing people with cancer…She was being treated in a hospital in Mexico City, but she abandoned her treatment and instead went to see the healer. She died a year later…”* (Cecilia, 28 years old).

##### Fear of COVID

Our participants reported that during the pandemic they avoided going to healthcare facilities because of fear of getting infected and dying of COVID. In addition to this postponement of health service utilization due to fear of COVID, they also reported difficulties to access health services due to the reconfiguration of healthcare services to prioritize attention for COVID. Those who tried seeking care faced even longer waiting times than usual to get consultations and tests.

*“…Now with the COVID pandemic it is more difficult going to the health center, people ask you “what are you going for?... you will get infected”.* (Patricia, 48 years old).

“…*So, they gave me the appointment a year and a half later, it took a year and a half for me to see the specialist. When I went a year and a half later, they told me “You need recent studies” and then they sent me to do a tomography a month later, then with the COVID pandemic and restrictions they have not given me the results of the tomography, and until know I’m still waiting to get attention…”* (Cecilia, 28 years old).

#### Health services organization

The majority of the perceived barriers for BC early diagnosis described by our participants were at the level of the health system. According to the HCQ framework, quality healthcare should be safe, effective, patient-centered, timely, efficient and equitable. Our focus groups participants perceived quality problems in the public health services that they are entitled to use, and the problems they described were mainly related with disrespectful (instead of patient-centered), untimely, and inequitable care.

##### Discrimination/Mistreatment by health care personnel

Our study participants reported experiences of disrespectful and even discriminatory treatment in their interactions with healthcare personnel in public services. They shared several personal experiences of abuse by healthcare personnel in public services. They questioned the reasons for this, and explained that they think it is due to a combination of their low levels of education, being women and being indigenous.

*“There are many times that I don’t know if it’s because of their profession or because they feel superior to us, they treat us badly. I mean, I have felt abuse, we all have experienced that, doctors even make fun of us…for example, when my children were born, they examined me, but it was a horrible examination, I mean they put their hands inside, they laughed…they even made fun of me. So I ask myself why? Why do they treat us like that?”* (Margarita, 38 years old).

##### Lack of trust in health personnel

Many of our participants expressed a lack of trust in doctors and healthcare personnel in general due to these past personal negative experiences as well as stories they have heard from other people in their community. For this reason, they try to seek care in private services which they perceive as better quality. The problem is that they often can’t afford it.

In more extreme cases, our participants described being denied healthcare. The health workers would tell them to return to their homes without giving them care. They would be told that it was due to administrative issues, or lack of time, or insufficient doctors, or sometimes without any explanation. This was perceived by our participants as “unfair” treatment.

*“I went for a consultation and they told me “come back in 8 days” so, the truth is, I was really upset because I really needed the service. But no, they told me to return in 8 days, so I wanted to report them for the bad attention, not just for me, but for the others because I have heard other people’s experiences. The truth is that it is unfair, they should work and serve with joy because they receive a salary”* (Nancy, 50 years old).

##### Language barriers

They also commented that language is a barrier for indigenous people who don’t speak Spanish. This mainly affects the elderly. Our participants expressed that healthcare personnel get angry when women don’t speak Spanish.

*“In the health center they don’t speak Otomí, if you speak in Otomí, they get angry, because they don’t understand…for example, there are elderly people who speak perfect Otomi, and they have to go with someone to translate, doctors say “Oh I didn’t understand you, a family member must come with you to translate”. They get angry”* (Patricia, 48 years old).

##### Long waiting times/Difficulties in making an appointment

Our participants described long times to get medical appointments at the local health center, long times to get referred to specialists, to receive test results and long hours waiting at the clinics to receive medical attention. They also described very complex administrative procedures to receive care, like having to arrive very early in the morning to the clinic and then stand in line for several hours in an attempt to get a medical consultation, without guarantee that they would succeed.

*“You have to go very early to get a voucher so that you can receive a consultation and you have to see if there are enough vouchers, because sometimes they just give a limited amount and if you don’t get one you have to go the next day and the next day to try to get one”* (Sonia, 42 years old).

*“…I had to go to the emergency room and that’s when they treated me. Then they told me “no, you have to go to your health center, and they have to give you a referral pass so that we can continue treating you”. So, I went back to my health center and they gave me the referral to the specialist a year and a half later…”* (Cecilia, 28 years old).

##### Costs/Distance to health services

Our participants described that financial barriers also limit their access to healthcare services, even if the consultations at public services are available without cost to the patient. Having to cover costs of medical care is not only a barrier for private service use. Our participants described that even if they manage to get a consultation in public services without having to pay, they often can’t cover the costs of the medicines that are prescribed.

*“I wanted to go back to the hospital, make my appointment again, but many commented that now you have to pay, many are commenting that now you have to pay even for the medicine”* (Alejandra, 42 years old).

In addition to direct medical costs, there are costs related to transportation and time. Some participants explained that the people in the community have to travel long distances and take several means of transportation to get to medical services, especially if they need specialized care.

*“To get there we go in public transport, in a community taxi, for example to get to the Jiquipilco hospital, we have to go up the hill and from the hill we have to transfer to another community)* (Nancy, 50 years old).

#### Interpersonal barriers

##### Influenced by peers

At this level of the Social Ecological Model, the influence of peers and family came up as very relevant in the decision of whether or not to seek care, when to seek care, and what type of care to seek: whether traditional medicine, or the local public health center, or even private services. It was reported that when women are ill, instead of going to the doctor, family and friends recommend treating with natural remedies, even one participant reported that a woman in the community with BC abandoned cancer treatment for traditional medicine on the recommendation of her husband.

“*And sometimes, as we said before, the opinion of the husbands, of the family, influences the women too much, it really influences them a lot… maybe they want to go to the doctor, but if they are told “oh, don’t go , there is a neighbor who was cured with such thing (natural medicine), take this, go with a “healer”” so I think they don’t go to the doctor because of that”* (Yolanda, 26 years old).

#### Individual barriers

##### Lack of cancer awareness

There was in general low cancer awareness among our participants. Even though they had heard about BC, they recognized they did not have enough information about the disease, its risk factors and how to diagnose it early.

*“I think that although we have heard about BC, we need a lot of information, especially in BC, because for example, the test for cervical cancer is much more feasible, we know about the Pap smear”* (Alejandra, 42 years old).

*“They have told us to explore our breasts ourselves, but how do we have to check them? We practically don’t know, maybe we touch a deformity but we don’t know if it is dangerous or not”* (Teresa, 47 years old).

##### Low risk perception of breast cancer

Although participants know other people who have been affected with BC, or have heard about it, some participants perceived themselves as not being at risk of developing BC. The fact of thinking that BC is mainly transmitted through family inheritance, makes them feel at low risk of developing it. In words of a participant *“I am certain that I will not develop that disease because no one in my family has had it”.* (Patricia, 48 years old).

##### Fear of cancer

Among our participants, fear of having cancer was perceived as an important barrier to seek care. They described that the fear of having the diagnosis confirmed could cause women in their community to postpone health care-seeking for breast symptoms. This fear is related with their fatalistic attitudes towards cancer.

*“Because yes, fear usually paralyzes you, right? You say “Oh no, maybe I feel something and I’m imagining the worst…you don’t want to know…there are many people who, despite the fact that maybe it is something very simple, find it very difficult to go to the doctor. Yes, going for treatment, going for a check-up, maybe it is not serious, but maybe they are already thinking that it is something fatal”* (Margarita, 50 years old).

### Perceived facilitators to early BC diagnosis

#### Social cultural level

##### Information by media

One of the elements that were found within the cues to action dimension were the messages and information received through the media (radio and television commercials), social networks, and screening mammography promotion activities done in their communities. They perceived all this information as facilitators for early breast cancer diagnosis. They find informative posters in the community and community health workshops very useful to keep themselves informed and to inform younger people on the importance of taking care of their health.

#### Health services organization

##### Respectful patient-centered medical care

One of the main perceived facilitators that women emphasized would facilitate early cancer diagnosis and medical attention of any health problem was receiving respectful, empathetic care with good attitudes of healthcare personnel and effective communication between doctors and patients. This was more aspirational than actual experiences of the participants.

*“It is very important that when we go to the health center we can be served, and that the doctors tell us what we should do, and it is also very important to be treated nicely so that we can trust doctors, and we are able to talk to them about what we feel or need.”* (Alejandra, 42 years old).

##### Information by doctors and promoters

Women reported receiving information about BC by health care personnel in public and private services. They had heard about breast self-examination mainly in public primary care clinics through nurses “We need a lot of talks, but I would like to include young people because they are beginning to take care of themselves so that they know the care they should have is very important” (Verónica, 46 years old).

### Interpersonal facilitators

#### Social support

*Social support* from other women and from their family members, especially their partners was reported as a potential key facilitator. Women shared that hearing experiences of women who had cancer could be a strong motivator for them to check themselves and go to the doctor. They also commented that the support of other women is key, especially in two ways: by accompanying them to the health center and by being able to share and discuss these issues with them.

*“It is important for women who have had it (cancer) that they talk about their experience because sometimes we see it on television but it is not the same, but if you know someone who had it and she talks about their story, how they lived it, that makes us more aware”* (Maribel, 35 years old).

#### Individual facilitators

##### Perceived severity of breast cancer

The fact that women perceived BC as a serious disease that begins without symptoms, progresses over time if women do not receive medical attention, and that can spread to other parts of the body and cause death, can motivate them to look for medical care.

“*Um, cancer are tumors that are in your body, something dark that grows inside you and can contaminate your entire body”* (Participant, 47 years old)

“*You have to go to the doctor, because we always leave it for later, but then, sometimes, with the passage of time, and when you want to go to the doctor, well... it’s too late, it turns too complicated for you and that’s it”* (María, 52 years old).

##### Perceived benefits of early BC diagnosis

Almost all participants were aware of the importance of cancer early detection. They mentioned that early detection increases the chances of cure, and that this motivates them to keep themselves informed and to talk about it with their peers.

## Discussion

This is the first study to explore perceived barriers and facilitators to timely healthcare seeking and access for early diagnosis of BC among Otomí indigenous women in Mexico. The results reveal barriers and facilitators at different levels of the Social Ecological Model that may inform interventions to improve early diagnosis of BC in this vulnerable population. Among the most salient barriers were: the elimination of well-established social programs that facilitated access to healthcare, fatalistic cultural beliefs about cancer, cultural gender roles related with prioritization of the care of other people, sexual taboos that can interfere with self-detection and healthcare seeking for breast symptoms, lack of trust in healthcare providers due to past experiences of mistreatment and discrimination, and access barriers for use of healthcare services.

One of the most striking findings of this study are the participants’ descriptions of mistreatment by healthcare personnel that they have experienced when using medical services. These seem to be a consequence of healthcare ethnic and gender discrimination. Although until recently healthcare racism towards indigenous people was overlooked, both in academia and public policy [59], there is emerging scientific evidence that identifies various forms of discrimination as a structural determinant of the lack of access to healthcare for these populations [60–62]. Healthcare personnel may hold unconscious biases and heuristics based on gender and ethnic stereotypes [63], that can negatively impact patient care [64]. These biases have been found to be further compounded when healthcare providers are faced with patients who are not only women but are additionally poor, from a rural community, and belong to a marginalized ethnic group [65]. The lack of physician cultural competency and implicit bias by clinicians toward ethnic, racial and gender minorities have been shown to result in the provision of unequal healthcare and disparities in cancer outcomes [66]. In turn, these experiences of mistreatment and discrimination, damage the patients’ trust in healthcare providers, and thus, can act thereafter as barriers to participation in screening, timely healthcare seeking for cancer symptoms and adherence to treatment [67–71].

The preference of traditional medicine over formal medical care services that is described by some of the study participants could be related, in addition to cultural health beliefs, to the mistreatment that indigenous people often experience when seeking medical care. The use of traditional medicine and home remedies has been described in other studies as a barrier to healthcare seeking of formal medical services and cancer awareness in other indigenous populations in Mexico and Ghana [72–74]. It has been described that they usually consult a traditional healer as a first point of contact, they believe that traditional healers have supernatural powers they have inherited from their ancestors, which cements their authority in the community [73]. Indigenous people have more trust in traditional medicine and traditional healers than in modern western medicine and medical care providers [75–77], although anthropological evidence allows us to recognize that they also value and make use of allopathic medicine [78]. Indigenous populations throughout the world have used traditional medicine for many generations, and many communities perceive it as valuable, affordable, and more acceptable as it aligns with their sociocultural beliefs [79–81]. In contrast, indigenous population have described western medicine as very impersonal with very short consultations, little space and opportunity to express their concerns, and almost no explanations of their illness [77]. However, criticism is directed primarily at how they are treated by healthcare personnel, not at the effectiveness of western medicine therapeutic resources. Also, access to traditional healers is easier for indigenous people both in terms of geographic proximity and waiting times to get a consultation. In addition, within these relationships with traditional healers there are no forms of discrimination and racism based on ethnic differences [75, 82].

Our participants described that they perceived easier access to formal healthcare services when the social program POP (Progresa-Oportundiades-Prospera) was in place. That program was coupled to health promotion and prevention activities that took place in health centers. After the elimination of this program in 2019, our participants describe that they lost the direct link they had to healthcare facilities where they could seek care. The elimination of successful social and health programs has also been described as an access barrier for use of reproductive health services by indigenous women in other studies [83]. The COVID-19 pandemic was a global health crisis that generated uncertainty and fear around the world. Learning and social interaction are factors that help us to understand how risk awareness and fear are generated in the presence of a pandemic [84]. Our results show that fear of becoming infected with COVID-19 acted as a barrier to approaching health centers. This is consistent with other studies, where COVID-19 fatality rates were higher in indigenous population in comparison to the rest of the Mexican population [85]. Additionally, due to the pandemic, many health centers were converted to only attend COVID-19 cases, while others were saturated, and this complicated access to the early diagnosis and treatment of cancer worldwide [86–88].

Another group of salient barriers identified in this study were cultural beliefs and roles: fatalistic cultural beliefs about illness, cancer stigma, gender roles related with prioritization of the care of other people, and sexual taboos that can interfere with the detection of breast symptoms and healthcare seeking for symptoms. The study participants described a widespread cultural belief among *otomíes* of cancer being seen as a divine punishment for “bad behavior”. Similar beliefs have been reported for African American and Hispanic women residing in the USA [89]. Seeking for healthcare is likely to be postponed if a person doesn’t believe there is much she can do to influence her health [89, 90]. Our study participants also described that a commonly shared belief in their community is that if a person thinks about an illness, he/she may attract such illness. This can also act as a barrier to preventive and healthcare seeking behaviors, as people opt to avoid thinking about any diseases in order to avoid being affected by them. To our knowledge, this belief has not been described in previous studies, but given its relevance, should be intentionally explored in future studies.

Another salient cultural belief that our participants described as having an impact on health behavior of women in particular is that of sexual taboos and embarrassment to touch their own bodies or have healthcare professionals examine their bodies. They see the touching of their own breasts as a sexual behavior that is disapproved in their community, especially by the men. In the same line, the male partners disapprove of their wives having their breasts or sexual organs examined by a doctor, especially if it is a man. These sexual taboos and male control over their female partners’ health and sexuality can act as barriers for early discovery of breast symptoms as well as for early seeking of medical care, as it has been reported for other populations [91, 92]. Embarrassment to be seen or touched by healthcare personnel has also been reported in the literature as a barrier for not participating in BC screening programs in other countries [93].

Finally, the main barriers identified at the individual level were limited cancer awareness -with misinformation about the disease, its risk factors and how to detect it early- and fear of being diagnosed with BC. Limited cancer awareness has been documented as a major barrier to seeking care, using medical services, as well as late detection and poor outcomes [16, 17, 20, 94, 95]. To increase individuals’ knowledge, awareness, risk perception and motivation to seek healthcare, educational interventions can be effective [96]. But, if they are to be effective in specific indigenous populations, the design of these educational interventions need to be tailored according to the needs, beliefs and cosmovision of the indigenous population towards which they will be directed to [97]. In addition, interventions directed to increase the perception of severity of the disease should simultaneously increase the perception of benefits of early diagnosis so that fear does not stop women from seeking care.

This study has some limitations. Due to our qualitative design and purposeful sampling strategy, our findings are not generalizable to the entire *otomí* population, not even that residing in Jiquipilco. Also, even though our study participants were instructed to speak on behalf of cultural views that would be representative of their communities, they may also have provided personal views. However, we believe this information is valuable as personal views are often a reflection of shared cultural values.

## Conclusions

This study identified barriers and facilitators for early diagnosis of BC as perceived by *otomí* indigenous women. Healthcare providers and policy makers should take notice of indigenous women’s beliefs, access barriers and healthcare discrimination experiences in the design of programs that aim to facilitate early BC diagnosis and treatment for these vulnerable populations. It is urgent to improve the quality of care and access to public healthcare services available in Mexico for the poor, especially for health problems where access to early diagnosis and treatment is key for good outcomes as is the case of cancer. Indigenous women, in addition to often being poor, too frequently face discrimination by healthcare providers due to their gender and ethnicity. Thus, beyond cultural differences, discriminatory treatment stands as a structural barrier to otomí women’s access to BC screening services. This is a characteristic shared by other Amerindian indigenous groups of people. Measures to prevent and eradicate all forms of mistreatment and discrimination in healthcare services are imperative.

## Data Availability

The data (de-identified interview transcripts in Spanish) that support the findings of this study are available on request from the corresponding author [KUS].

## Abbreviations

BC: BC
LMICs: Low- and middle-income countries
HICs: High-income countries
CEDIPIEM: The State Council for the Integral Development of Indigenous People (for its acronym in spanish)
SEM: Social Ecological Model
HBM: Health Belief Model
HCQ: Healthcare Quality
POP: “*Progresa-Oportunidades-Prospera*” (A social program in Mexico)
MST: Minerva Saldaña Téllez
KUS: Karla Unger Saldaña
